# Effects of Heat Stress in Dairy Cows Offered Diets Containing Either Wheat or Corn Grain during Late Lactation

**DOI:** 10.3390/ani12162031

**Published:** 2022-08-10

**Authors:** Josie B. Garner, S. Richard O. Williams, Peter J. Moate, Joe L. Jacobs, Murray C. Hannah, Greg L. Morris, William J. Wales, Leah C. Marett

**Affiliations:** 1 Agriculture Victoria Research, Ellinbank, VIC 3821, Australia; 2 Centre for Agricultural Innovation, School of Agriculture and Food, Faculty of Veterinary and Agricultural Sciences, The University of Melbourne, Parkville, VIC 3010, Australia

**Keywords:** heat stress, milk production, ruminant nutrition, dairy cows

## Abstract

**Simple Summary:**

A common nutritional strategy to reduce heat stress on dairy cows is to provide a more slowly degradable starch source that reduces the amount of heat generated during digestion. The aim of this experiment was to investigate the responses of late lactation dairy cows to cereal grain-based diets in a short-term heat challenge. Cows were offered a diet of alfalfa hay supplemented with either wheat grain (fast rumen degradable) or corn grain (slow rumen degradable). Individual cow measurements of feed intake, milk yield and composition, respiration rate, and body temperature were taken daily before, during and after a 4-day heat challenge, during which the cows were in individual controlled-climate chambers and exposed to air temperature up to 33 °C with 50% relative humidity. While exposed to the heat challenge during late lactation, cows that were offered corn grain had greater feed intake and tended to produce more energy-corrected milk but had lower respiration rates and similar body temperature to the cows offered wheat grain. The economic impact of feeding corn in place of wheat grain needs to be assessed before any comparative value of feeding corn grain or wheat grain can be determined.

**Abstract:**

Cereal grains that differ in the rate and extent of ruminal fermentation differ in heat increment and may be used to improve thermoregulation during heat stress. This experiment investigated the responses of dairy cows in late lactation to a heat challenge when offered wheat-grain or corn-grain. Eighteen lactating cows, 220 ± 94 (mean ± standard deviation) days in milk, 3.7 ± 0.17 years of age and 558 ± 37 kg bodyweight, were allocated treatments containing 6 kg dry matter (DM)/day of wheat grain or 6 kg DM/day corn grain (9 per treatment) plus 14 kg DM/day of alfalfa hay. Measurements were made during a 7-day pre-challenge period at ambient conditions in individual stalls, during a 4-day heat challenge (temperature humidity index of 74 to 84) in individual controlled-climate chambers, then during a 7-day recovery period at ambient conditions in individual stalls. During the heat challenge, cows offered corn had lower respiration rates (*p* = 0.017) and greater feed intake (*p* = 0.021) but energy-corrected milk (*p* = 0.097) was not different to that of cows offered wheat. Feeding corn grain to dairy cows during a heat challenge reduced some of the negative impacts of heat stress, enabling the cows to consume more forage compared with supplementing with wheat grain.

## 1. Introduction

High-producing dairy cows are particularly susceptible to heat stress [1] with responses including elevated body temperature, respiration rate and panting score and decreased feed intake and milk production. The temperature and humidity index (THI) threshold for heat stress is generally considered to be 68 to 72 [2] with production losses noted between 21 and 25 °C [1], depending on relative humidity. The effects of heat stress can be ameliorated to some degree by shade, sprinklers, fans and adequate water [2]. However, these strategies may not be as effective in Australian pasture-based dairy systems as the cows spend much of their time grazing compared with housed cow systems. Complementary strategies such as reducing the heat load on dairy cows in addition to shade may provide further benefits for grazing cows [1,2].

Fermentation in the rumen creates heat, which contributes to the overall metabolic heat production [3], but feed types produce different amounts of heat. For example, the heat of fermentation may be reduced by the inclusion of more slowly fermenting starch such as corn instead of more quickly fermentable starch such as wheat grain, which is commonly fed to grazing cows [4,5,6]. When corn grain is offered to ruminants, up to 40% of the starch can escape ruminal fermentation due to its slower rate of degradation in the rumen [7], and the escaped starch is subsequently fermented into simple sugars in the small intestine. In contrast, much of wheat starch is fermented in the rumen, leading to a theoretically greater amount of heat production than from corn [7]. This is supported by an experiment that measured the left and right flank surface temperature of dairy cows under thermoneutral conditions. Cows offered corn had lower left and right flank temperatures than those offered wheat [8]. These results suggest that heat of fermentation and metabolic body heat may be lower when cows are offered diets containing corn instead of wheat.

Wheat is the cereal grain most commonly offered to dairy cows in Australia [9,10]. Increased ruminal temperatures coinciding with periods of heat stress may cause a reduction in dry matter intake (DMI) [11], accounting for some loss of milk yield [12,13]. In southern Australia from 2000 to 2008, the average number of consecutive days with a mean daily temperature and humidity index (THI) greater than 75 was four [14]. This duration of heat exposure can be considered short-term based on previous reports that suggest there are different production responses in lactating cows exposed to short-term heat exposure of two to four days [15,16] compared with longer-term heat exposure of seven or more days [12]. Hou et al. [17] carried out a direct comparison of short-term (3 d) and long-term heat exposure (7 d) and reported a greater reduction in milk yield in cows exposed to long-term heat stress. The objective of this experiment was to measure feed intake, milk production, body temperature and respiration rate of late-lactation dairy cows offered diets containing either wheat or corn grain during periods of ambient conditions and during a short-term, 4-day heat challenge.

We hypothesized that during a short-term heat challenge, cows offered a diet containing corn grain during late lactation would have (1) greater DMI and energy-corrected milk (ECM) production and (2), a lower body temperature than those offered a diet containing wheat grain.

## 2. Materials and Methods

### 2.1. Cows and Design

Eighteen second-lactation, pregnant Holstein–Friesian cows were offered one of two dietary treatments. The experimental animals were 3.7 ± 0.17 (mean ± standard deviation) years of age, 20.7 ± 2.4 kg/day milk yield, 101 ± 4.8 genomic estimated breeding value for heat tolerance (GEBV, DataGene ABV issued July 2020, datagene.com.au), 220 ± 94 days in milk (DIM), 558 ± 37 kg bodyweight (BW), 4.8 ± 0.22 body condition score on the 1 to 8 scoring system of Earle [18]. The dietary treatments were: (1) (CRN) basal diet plus 6 kg DM/day of crushed corn grain, or (2) (WHT) basal diet plus 6 kg DM/day of crushed wheat grain. The basal diet consisted of 14 kg DM/day of chopped alfalfa hay, 42 mL/day bloat drench (Bloat Drench; VicChem, Coolaroo, VIC, Australia), 100 g/day salt, and 200 g/day minerals: Ca 134 g/kg, Mg 110 g/kg, P 60 g/kg, Zn 6.4 g/kg, Mn 2.4 g/kg, Cu 1.2 g/kg, I 80 mg/kg, Co 100 mg/kg, Se 24 mg/kg, Vitamin A 165 IU/g, Vitamin D3 24 IU/g, and Vitamin E 800 mg/kg. The chemical compositions of the main dietary ingredients are shown in Table 1.

Cows were divided into 3 cohorts of 6 such that cohort 1 comprised the 6 cows with the lowest bodyweight, and cohort 3 comprised the 6 cows with greatest bodyweight. Each treatment was allocated at random to 3 of the 6 cows within each cohort taking into account balanced milk yield, DIM, and GEBV for heat tolerance. This was achieved using the COVDESIGN procedure in GenStat 18 (VSN International Ltd., Hemel Hempstead, UK). Within a cohort, cows were randomly allocated to one of six controlled-climate chambers.

The experiment consisted of five periods: a 7-day covariate period (ambient conditions, individual feed stalls), a 7-day diet adaptation period (ambient conditions, individual feed stalls), a 7-day pre-challenge period (ambient conditions, individual feed stalls), a 4-day heat challenge period (diurnal variation in THI of 74 to 84, cows in individual controlled-climate chambers), and a 7-day recovery period (ambient conditions, individual feed stalls). During the covariate period, each cow was offered 14 kg DM/day of chopped alfalfa hay and 6 kg DM/day of equal quantities of corn (3 kg DM) and wheat (3 kg DM) grain for 7 days in individual feed stalls. During the 7-day adaptation period, cows were transitioned and adapted to their treatment diets in individual feed stalls, reaching their full treatment ration by day 7 of the adaptation period. During the 7-day pre-challenge period, cows were offered their treatment diets in individual feed stalls. Cows were then moved to individual controlled-climate chambers (No Pollution industrial systems, Edinburgh, UK), where they were exposed to the heat challenge for 4 days following protocols developed by Garner et al. [19]. They were offered treatment diets and milked in situ in the chambers for 4 consecutive days. After the heat challenge, cows were returned to the individual feed stalls and loafing area for the 7-day recovery period.

### 2.2. Experimental Procedures

The cows were trained in, and adapted to, the individual feed stalls and controlled-climate chambers before the experiment. During the covariate, adaptation, pre-challenge and recovery periods, cows were allowed 3 h after the AM milking and 3 h after the PM milking to consume their ration in the individual feed stalls and have access to water and were then returned to a loafing area with soft bedding and access to water ad libitum.

The conditions in the controlled-climate chambers for the heat challenge were designed to remain above THI 74, but not exceed THI 84, to impose a mild to moderate level of heat stress [20]. The climatic conditions programmed were 25 °C and 60% RH (THI 74) between 18:01 and 06:00, 30 °C and 50% RH (THI 80); and between 06:01 and 12:00, and 33 °C and 50% RH (THI 84) between 12:01 and 18:00. The cycle of 12 h light (06:00 to 18:00) and 12 h dark (18:00 to 06:00) was controlled manually.

If an individual cow’s rectal temperature was greater than 40.9 °C, the cow was cooled by opening the chamber doors, adjusting the conditions in the chamber to thermoneutral (17 °C, 60% RH) and intermittently wetting the cow with cool water until her temperature was less than 40.1 °C. Cows thus cooled were removed from the chambers and returned to the feed stalls where they were managed as previously described.

### 2.3. Measurements and Sampling

Grain and alfalfa offered and refused were recorded twice daily during all experimental periods. Samples of feed offered and refused were collected twice daily, for dry matter and analysis of chemical composition. Samples were freeze dried, ground to pass through a 0.5 mm screen and analyzed for DM, crude protein, neutral detergent fiber, acid detergent fiber, lignin, ash, starch with estimated metabolizable energy subsequently calculated [21,22]. Gross energy was calculated using the approach of Atwater and Woods [23].

Cows were milked twice daily at approximately 06:00 and at 15:00 with the yield recorded automatically in the milking parlor at each milking during all periods except during the heat challenge (MM25; DeLaval International, Tumba, Sweden). During the heat challenge period, cows were milked using the built-in milking system (same clusters and pulsators as in the milking parlor), and yields were measured manually by weighing. Milk composition (fat, protein, lactose) was analyzed by a near-infrared milk analyzer (Model 2000, Bentley Instruments, Chaska, MN, USA). Energy-corrected milk was calculated using the equation of Tyrrell and Reid [24] as implemented by Moate et al. [25].

Throughout the experiment, except during the heat challenge period, individual cow bodyweights were recorded daily after each milking using walkover scales (AWS100; DeLaval, Tumba, Sweden).

During the covariate period (days 1 to 7), bodyweight, feed intake and milk yield were measured daily; milk composition was measured on two separate days; respiration rate was measured a.m. and p.m. on one day; and vaginal temperature was recorded continuously for 5 days.

During the adaptation period (days 8 to 14), cows were transitioned to the diet and bodyweight, feed intake and milk yield were measured daily; milk composition was measured on two separate days; and respiration rate was measured a.m. and p.m. on one day.

During the pre-challenge period (days 15 to 21), individual cow bodyweight, feed intake and milk yield were measured daily and samples for milk composition collected over three days. Respiration rate was measured a.m. and p.m. on two days and vaginal temperature was recorded continuously for 5 days. Blood samples were taken on day 21.

For the heat challenge period (days 22 to 25), cows were housed in individual controlled-climate chambers. Individual cow feed intake and milk yield were measured with samples for milk composition collected a.m. and p.m. daily. Respiration rate, panting score and rectal temperature were measured a.m. and p.m. daily, and vaginal temperature recorded continuously for the 4 days of the heat challenge. Blood samples were taken on day 25.

For the recovery period (days 26 to 32), cows were returned to the individual feed stalls and loafing areas. Individual cow bodyweight, feed intake and milk yield were measured daily with samples collected for milk composition a.m. and p.m. on three separate days. Respiration rate was measured a.m. and p.m. on two days and vaginal temperature was recorded continuously on days 26 to 31. Blood samples were taken on day 28.

Ambient temperature and relative humidity during the covariate, adaptation, pre-challenge and recovery periods were measured using a weather station every 10 min (Model J3504; Measurement Engineering Australia, Magill, SA, Australia) located 850 m northwest of the feeding facility.

### 2.4. Physiology

On measurement days after milking, cows were moved to a race and allowed to settle for at least 10 min. Respiration rate (breaths per minute) was then assessed by counting visible flank movements, and panting was scored during the heat challenge according to the procedure of Gaughan et al. [26]. Rectal temperature was then measured using a large-animal digital thermometer (model 212771; Shoof International, Cambridge, New Zealand). Vaginal temperature was recorded using modified intravaginal CIDR (Zoetis, Melbourne, VIC, Australia) housing temperature loggers (iButton DS1922L; Thermodata, Warrnambool, VIC, Australia) every 10 min.

Blood samples from each cow were taken at approximately 14:45 via coccygeal venipuncture on day 7 of the pre-challenge period, day 4 of the heat challenge period, and day 3 of the recovery period. Using a vacutainer containing potassium EDTA (BD Vacutainer System, Plymouth, UK) 10 mL blood samples were taken. Using a syringe, approximately 0.1 mL of blood was removed immediately after collection and inserted into the reader chip of a self-calibrating blood gas analyzer (Epoc Host2 Zebra MC55A0; Epocal Inc., Ottawa, ON, Canada) and test cards (epoc BGEM; Epocal Inc., Ottawa, ON, Canada) as per the manufacturer’s instructions. The analytical variables determined were partial pressure of carbon dioxide (pCO_2_), partial pressure of oxygen (pO_2_), sodium (Na^+^), potassium (K^+^), chloride (Cl^−^), glucose, and lactate; and the calculated values of bicarbonate (cHCO_3_^−^) and total carbon dioxide (cTCO_2_).

### 2.5. Calculations and Statistical Analyses

Milk records were matched with feed intake such that the a.m. and p.m. feed intake of one day were matched with the p.m. milk recorded for that same day and the a.m. milk the following day. This was to allow for the delay between feed being consumed and used.

The temperature–humidity index was calculated using Equation (1) [27].

THI = Tdb + (0.36 × Tdp) + 41.2(1)

where: Tdb = hourly dry bulb temperature (°C); Tdp = the dew point temperature (°C); Tdp = (237.3 × b)/(1.0 − b); b = (log(RH/100.0) + (17.27 × Tdb)/(237.3 + Tdb))/17.27; and RH = relative humidity (%).

Duration of vaginal temperature higher than 38.8 °C was calculated as the number of minutes that it exceeded this threshold on a given day (06:00 to 06:00).

Two types of statistical analyses were conducted. The first tested the heat challenge to see if it influenced major variables, while the second examined dietary treatment effects in each of the experimental periods, or the changes between periods. Variables were constructed to address hypotheses, with one datum per animal. To achieve this, raw data were averaged within each of the 7-day covariate, 7-day pre-challenge (day 7 for blood analytes), 4-day heat treatment (day 4 for blood analytes), 7-day recovery periods, and on day 7 of the recovery period (day 3 for blood analytes and day 6 for VT due to 06:00–06:00 daily measures) for each animal. Respiration rate and panting score were averaged within a day before being averaged within a period.

The first type of statistical analysis was performed on the major physiological indicators of heat stress (DMI, milk yield, ECM yield, body temperature, respiration rate and blood analytes), and it tested if the means of specific variables differed between periods. This was achieved by the use of paired Student’s *t*-tests of data from two periods. Standard errors of differences between means (SED) and *p*-values were calculated for the following contrasts: Pre-challenge to heat challenge, heat challenge to recovery and pre-challenge to recovery.

The second type of statistical analysis examined dietary treatment effects during each experimental period. For these analyses, additional variables were calculated. Rates of change per day during the heat challenge and the recovery period were calculated for each cow as regression slopes using a median linear regression of daily data versus day and calculated by the RQLINEAR procedure in GenStat 21, (VSN International Ltd., Hemel Hempstead, UK). For change per day during heat stress, the regression data included the final day of the pre-challenge and the four subsequent days under heat stress. For the change per day during recovery, the regression data included the seven recovery days. Each of these constructed variables (the period means, changes in period means, the rates of change during the heat challenge and recovery periods, and data on the selected days) was subjected to statistical analysis by analysis of covariance (ANCOVA). The treatment structure for the ANCOVA was diet, and the blocking structure was the cow nested within the cohort. The model included a linear covariate, if available, corresponding to the measurement type, taken during the covariate period.

*y*_*ij*_ = *μ* + *βx_ij_*+ *D_d(i,j)_* + *C_j_* + *ε_ij_* (2)

where *y_ij_* was the outcome variable for cow *i* in cohort *j*; *μ* was the mean; *x_ij_* was the measurement from the covariate period (if available), centered, with coefficient *β*; *D_d(i,j)_* was an effect of the diet *d_(i,j)_* given to cow *i* in cohort *j*; *C_j_* was an effect of cohort *j*; and *ε_ij_* the residual error associated with each cow. All statistical analyses were conducted using GenStat 21. Residuals were examined graphically to check distributional assumptions of normality and constant variance. *p*-values of < 0.05 were considered significant, and those ≥0.05 and <0.10 were considered a trend.

Four cows were excluded from the analysis. Three (2 CRN, 1 WHT) did not complete the heat challenge due to exceeding the predetermined body temperature threshold, and one (WHT) was removed due to an unrelated illness.

## 3. Results

**Figure 1:**
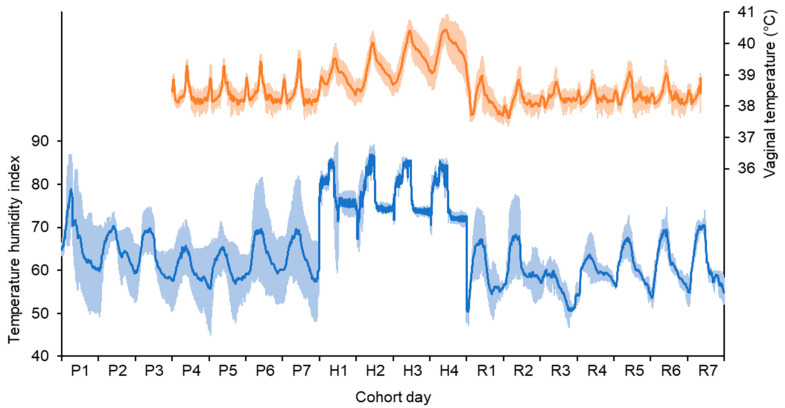
Mean environmental conditions experienced by the cows before (P), during (H), and after (R) a heat challenge (blue line, light blue band shows ± one standard deviation about the mean) and the mean vaginal temperature of all cows (orange line, light-orange band shows ± one standard deviation about the mean). The heat challenge was generated in controlled-climate chambers, and the cows were kept in ambient conditions before and after the heat challenge.

During the experiment, the ambient weather conditions outside the controlled climate chambers were air temperature of 17.1 ± 5.54 °C (daily mean ± standard deviation), relative humidity of 75 ± 21.1%, and THI of 64 ± 6.8. During the heat challenge the cows experienced air temperature of 26.8 ± 3.55 °C, relative humidity of 58 ± 8.4%, and THI of 76 ± 4.5. The daily THI pattern experienced by the cows during the pre-challenge, heat challenge and recovery periods is shown in Figure 1.

The coefficient of variation in THI was 0.11 during the pre-challenge, 0.02 during the heat challenge, and 0.05 during the recovery period.

### 3.1. Main Effects of the Heat Challenge

**Figure 2:**
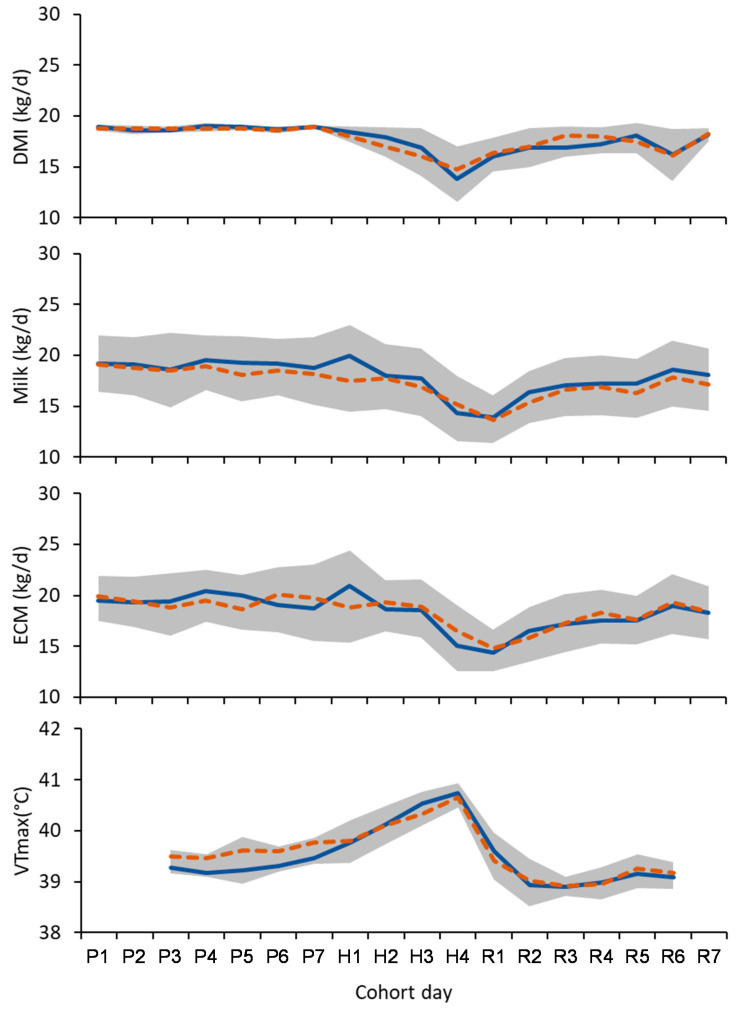
Mean daily dry matter intake (DMI), milk yield, energy-corrected milk yield (ECM) and maximum vaginal temperature (VTmax) of cows offered corn grain (solid blue line) or wheat grain (dashed brown line) before (P), during (H), and after (R) a heat challenge. The shaded grey band shows ± one standard deviation about the experiment mean. The heat challenge was generated in controlled-climate chambers, and cows were kept in ambient conditions before and after the heat challenge.

Daily changes in DMI, milk yield and ECM during the pre-challenge, heat challenge and recovery periods are shown in Figure 2. The heat challenge induced heat stress symptoms in the cows across both dietary treatments. This was evidenced by a reduction in mean daily DMI of 2.3 kg DM (±0.34 (SED), *p* < 0.001) and milk yield by 1.07 kg (±0.38, *p* < 0.013) during the heat challenge compared to the pre-challenge period. Mean respiration increased by 43 breaths per min (±3.2, *p* < 0.001); vaginal temperature increased by 1.06 °C (±0.09, *p* < 0.001), concentrations of fat in milk increased by 0.30 g/kg (±0.067 g/kg, *p* < 0.001) and lactose increased by 0.18 g/kg (±0.063, *p* = 0.011). There was no change in mean milk protein concentration (*p* = 0.912) or in mean daily ECM yield (*p* = 0.246) between the pre-challenge and heat-challenge periods.

A range of blood analytes was affected by exposure to the heat challenge. There were reductions in the blood concentration of pCO_2_ of 12.8 mmHg (±3.53, *p* = 0.002), cHCO_3_^−^ by 4.5 mmol/L (±0.45, *p* < 0.001), cTCO_2_ by 4.9 mmol/L (±0.55, *p* < 0.001), and K+ by 0.6 mmol/L (±0.27, *p* = 0.054) during the heat challenge compared to the pre-challenge period. There were increases in the blood concentrations of Na^+^ by 2.5 mmol/L (±0.48, *p* < 0.001), Cl^−^ by 4.1 mmol/L (±0.80, *p* < 0.001), glucose by 0.2 mmol/L (±0.05, *p* < 0.001) and lactate by 0.5 mmol/L (±0.19, *p* = 0.012) during the heat challenge compared to the pre-challenge period.

Recovery from the heat challenge was such that by day 7 post-heat challenge the DMI and milk yield of cows were not different from that measured during the pre-challenge period. Similarly, the concentrations of most of the analytes in blood sampled during the recovery period showed no difference. However, blood concentrations of cHCO_3_^−^, cTCO_2_ and lactate remained lower during the recovery period compared to the pre-challenge period (−1.6 ± 0.53, −1.6 ± 0.60 and −0.3 ± 0.13 mmol/L, respectively; *p* < 0.05).

### 3.2. Feed Intake

During the pre-challenge period, feed intake was stable with the pooled within-cow variance being 0.04 for DMI (Figure 2). Mean grain and forage intake, and total DMI between the cows offered the CRN and WHT diets showed no difference (Table 2). The ratio of forage to grain consumed also showed no difference from the treatments during the pre-challenge period.

During the heat challenge, the mean intake of dry matter from grain showed no difference (*p* = 0.441), but the mean intake of dry matter from forage (*p* = 0.016) and total DMI (*p* = 0.021) were greater for the cows offered the CRN diet compared to those offered the WHT diet, as was the ratio of forage to grain consumed: 1.95 vs. 1.68 ± 0.095, *p* = 0.018.

During the recovery period there was no difference in mean intake of dry matter from grain, but cows offered the CRN diet had a greater intake of forage (*p* = 0.042) and a tendency for greater total DMI (*p* = 0.061). The ratio of forage to grain consumed was also greater for the cows offered the CRN diet than for those offered the WHT diet (1.90 vs. 1.74 ± 0.061, *p* = 0.022). On day 7 of the recovery period, feed intake and the ratio of forage to grain consumed were not different to those measured during the baseline period and were unaffected by treatment.

During the pre-challenge, heat challenge and recovery periods, the mean intake of metabolizable energy and starch were greater (*p* = 0.002) for cows offered the CRN diet.

### 3.3. Milk Yield and Composition

During the pre-challenge period, milk yield and ECM yield were stable, with the pooled within-cow variance being 0.12 for milk yield, and 0.18 for ECM yield (Figure 2). Milk yield and composition were not different between dietary treatments during the pre-challenge period (*p* > 0.10; Table 3).

During the heat challenge, dietary treatment had no effect on milk yield (*p* = 0.125). However, there were trends for cows offered the CRN diet to have greater yields of ECM (*p* = 0.097), lactose (*p* = 0.098) and milk protein (*p* = 0.040) than those offered the WHT diet. Concentrations of fat, protein and lactose were not (*p* > 0.10) affected by dietary treatment.

During the recovery period, cows offered the CRN diet had greater mean yields of ECM (*p* = 0.005), fat (*p* = 0.004) and protein (*p* = 0.005), and a trend for greater mean yields of lactose (*p* = 0.062). During the recovery period, mean concentrations of fat and protein were not affected by dietary treatment, but the cows offered the CRN diet had a greater mean concentration of milk lactose (*p* = 0.019) compared to those offered the WHT diet.

On day 7 of the recovery period, milk production was similar to that measured during the pre-challenge period in both treatments. There was no difference in milk and ECM yields between treatments on day 7 of the recovery period (*p* > 0.01).

### 3.4. Physiology

The diurnal pattern of vaginal temperature averaged across all cows during the experiment is shown in Figure 1, and the mean daily maximum vaginal temperature for cows offered the CRN and WHT treatment diets are shown in Figure 2. During the pre-challenge period, there were no differences in vaginal temperature or respiration rate (Table 4).

During the 4-day heat challenge, there were no differences in vaginal temperature parameters (*p* > 0.17), but the cows offered the CRN diet had lower respiration rates (*p* = 0.017) (Table 4). The mean panting score was also lower in cows offered the CRN diet compared to those offered the WHT diet (0.9 vs. 1.2, SED = 0.1, *p* = 0.048).

During the 7-day recovery period, there was no difference in mean or minimum vaginal temperature, but there was a trend toward greater maximum vaginal temperature (*p* = 0.059) and respiration rate was lower (*p* = 0.039) in cows offered the CRN diet than those offered the WHT diet. On day 6 of the recovery period, vaginal temperature was similar to the pre-challenge period, and there was no effect of treatment.

During the experiment, there was no difference in blood analytes (Table 5; *p* > 0.05) except that during the pre-challenge period, the blood lactate concentration was lower (*p* = 0.039), and K^+^ concentration tended to be greater (*p* = 0.073) in cows offered the CRN diet. In cows offered CRN compared to WHT, there was a tendency for the blood concentration of Na^+^ to be lower (*p* = 0.081) during the heat challenge period, and for the concentration of K^+^ to be greater (*p* = 0.093) during the recovery period.

### 3.5. Rates of Change within Period

During the heat challenge, the rates of change in the DMI of forage (*p* = 0.034) and total DMI (*p* = 0.031) (expressed as a regression slope) and the rate of change in milk fat concentration (g milk fat/kg per day, *p* = 0.025) were less for the cows offered CRN than for the cows offered WHT (Table 6). 

During the recovery period, the rate of change of forage intake was lower for the cows offered the CRN diet (*p* = 0.029), but there was only a tendency for the rate of change in total DMI (*p* = 0.056) and the rate of change in milk yield (kg/d) (*p* = 0.074) to be lower (Table 6). The rates of change in milk protein yield (kg milk protein/day) (*p* = 0.025) and milk lactose yield (kg milk lactose/day) (*p* = 0.003) for cows offered the CRN diet were also lower than for the cows offered the WHT diet. (Table 6).

## 4. Discussion

The late-lactation cows in this experiment had lower DMI, body temperature and respiration rate during the acute heat challenge than during the ambient conditions of the pre-challenge period. The observed changes in DMI, milk yield and physiology responses are consistent with these cows experiencing heat stress [26] as intended. Our results are consistent with previous studies that also reported declines in DMI [19,28,29] and milk yield [13,19,30] between the pre-challenge and heat challenge periods. In our experiment, the concentrations of milk fat and lactose were greater during the heat challenge. The effects of heat exposure on the yield and concentration of milk fat, protein and lactose are equivocal in the literature. In agreement with our data, Garner et al. [15,19] reported an increased concentration of milk fat during a 4-day heat exposure period of THI up to 84. In contrast, Gao et al. [30] and Cowley et al. [28] reported no change in milk fat concentration during heat exposure periods of 9 and 7 days at THI of 84 and >78, respectively. Similarly, there are numerous contradictory reports of other milk components including protein and lactose [12,13,29]. Thus, the overall impact of heat stress on milk composition remains unclear and may be influenced by the severity and duration of heat exposure in addition to nutrition and the stage of lactation.

During the recovery period, both DMI and ECM increased with time while body temperature remained steady. However, despite the nadir in DMI occurring during the heat challenge period, the nadir in milk occurred on day 1 of the recovery period. The reason for this delay is not known, but we suspect it was due to the lag between feed intake and milk yield. A lag time of 12 h between feed intake and milk production had been allowed for in the feed and milk pairing, but it is possible that the lag time was greater during heat stress. Delays in responses to hot weather had been previously identified [31], and there are reports of the weather up to 4 days prior affecting milk yield [32]. Together, these observations suggest that the lag time between the onset of hot weather and visible effects is less for feed intake than for milk yield.

During the heat challenge, cows offered the diet containing corn grain had a greater total DMI than those offered the wheat grain diet. These data partly support our first hypothesis, and we acknowledge the low number of animals used in this experiment. The difference in total DMI between treatments was due to a difference in the intake of forage, as there was no difference in grain intake between treatments during the heat challenge. It is important to note that our cows were offered their grain before their forage. Grain and forage were not available at the same time, which mimicked the general feeding regimen observed on the majority of Australian dairy farms where grain is offered at milking times, and forage is offered afterwards. It may be that the cows stopped eating when they had consumed sufficient feed, and this happened to be while the forage was being offered. The reason that our cows that were offered the CRN diet had the greater intake of forage and total DM is not known with certainty. However, this difference could be due to a difference in intra-ruminal temperature. Increases in intra-ruminal temperature have been shown to result in decreases in forage intake [11,33]. Furthermore, cows consuming corn grain have been reported to have lower flank temperatures than cows consuming wheat [8], reflecting differences in intra-ruminal temperature caused by the different fermentation characteristics of the two grains [7,34]. Therefore, we speculate that the greater intake of forage and total DM by our cows offered the CRN diet was consistent with the CRN diet causing a lower intra-ruminal temperature compared to the WHT diet. Further research to assess the direct impact of feed on rumen temperature is indicated.

During the 7-day recovery period, the mean yields of ECM, milk fat and milk protein of the cows offered the CRN diet were greater despite a slower rate of intake recovery post-heat exposure compared to WHT cows (Table 6). The slower rate of recovery is likely due to the smaller decline in milk yield and DMI in cows offered CRN. The difference in ECM yield during the recovery period reflected the greater intake of forage by cows on the CRN diet compared to those offered the WHT diet during the heat and recovery periods. By day 7 of the recovery period, milk yield had essentially returned to baseline and there was no difference in yield between treatments. These data suggest a positive effect of feeding corn to lactating cows during hot weather based on ECM yield, which is closely linked to feed intake. Further investigation to confirm this is warranted, given the low number of cows in our experiment and the short duration of the heat challenge.

The respiration rate was lower in cows offered the CRN diet during the heat challenge compared with those offered the WHT diet, but body temperature was not different between treatments. Thus, we partially accept our second hypothesis. The body temperature of cows resulted from the net difference between heat load and heat loss. When the heat load was greater, body temperature rose [35]. While body temperature was not affected by treatment, the CRN cows were likely shedding less heat via respiration than the WHT cows. The total heat load on an animal comes from digesting feed and the environmental conditions during hot weather. Since the cows offered CRN consumed more feed than those offered WHT, it is possible that some of the expected differences in body temperature were offset by variations in the heat-generating properties of the feed during digestion. Corn grain may have a lower fermentation heat than wheat grain due to its slower rate of ruminal fermentation [36,37] and resulting volatile fatty-acid profile with a higher acetate-to-propionate ratio and thus lower thermogenesis [37,38,39]. Therefore, the CRN diet may have had a lower heat increment than the WHT diet. This is supported by the results of Gonzales-Rivas et al. [6], who reported that cows offered a TMR with corn grain had lower body temperature than cows offered the same TMR but with wheat. However, both our results and those of Gonzales-Rivas et al. [6] are in contrast to the findings of Garner et al. [15], who reported that cows fed corn grain had a greater body temperature than those fed wheat grain. These inconsistencies may be a result of variation in DMI, forage-to-concentrate ratio, stage of lactation, intensity and type of heat exposure (acute or chronic), or the manner of presenting the diet (total mixed ration or grain fed separately to forage). Additional research is required to understand how changing the type of concentrate offered to cows during hot weather affects body and rumen temperature.

Exposure to the heat challenge caused significant changes to the blood chemistry of cows. However, only minor differences between cows offered CRN compared to WHT were observed. Most of the blood analytes were consistent with previous reports, but the blood values for pCO_2_ and pO_2_ were greater than in previous reports of ruminants offered similar diets [40,41]. The reason for this is unclear, but may have resulted from taking the blood sample from the coccygeal vein rather than the artery [42] or from the confined space within the controlled-climate chambers. More consistent with previous reports [40] is that exposure to the heat challenge reduced pCO_2_, HCO_3_^−^ and TCO_2_, indicating hypercapnia. The blood concentration of Na^+^, Cl^−^, glucose and lactate were each increased during the heat challenge compared to the pre-challenge period. This contrasted with the results of Prathap et al. [40] in heat-stressed sheep offered diets containing wheat or corn. In their experiment Na^+^ in blood was increased during heat exposure, but Cl^−^, glucose and lactate were not changed. The only changes to blood analytes due to offering CRN to cows compared to WHT in our experiment was mainly during the pre-challenge period during which blood lactate concentration was greater and K^+^ concentration tended to be greater. The greater K^+^ concentration in ruminants fed diets containing corn was consistent with the results of Prathap et al. [40]. Despite the lower respiration rate in cows offered CRN compared to WHT, it did not ameliorate the changes in blood biochemistry induced by heat stress.

The decline in milk from the pre-challenge to the heat-challenge period appeared to be fully accounted for by the decline in metabolizable energy intake. The largest declines were observed in the cows offered the wheat diet where milk energy requirement declined by 9 MJ (~5MJ/L, [43]) but the intake of metabolizable energy declined by 30 MJ. This suggested that the cows must have used body reserves to support milk production during the acute heat challenge in our experiment. In contrast, it has been reported that the milk yield of cows in a heat challenge declined more than their pair-fed counterpart in thermoneutral conditions [12,28], indicating that the decline in feed intake did not account for all of the decline in milk yield. There are also examples where this was not the case [13]. We speculate that differences in diet formulation, feed presentation (components or mixed), and heat intensity and duration could all influence the mechanism that animals use to manage heat stress. The responses measured in this experiment may also have been influenced by the late stage of lactation. This is because the threshold at which heat stress begins has been shown to be lower for cows in early compared to late lactation, or for cows producing high (≥22 kg/day) compared to low amounts of milk (≤10 kg/day) [1]. Further research is necessary to untangle these complexities.

## 5. Conclusions

Feeding corn grain to dairy cows appears to reduce some of the negative effects of heat stress observed during an acute heat challenge compared to feeding wheat grain. During the heat challenge in our experiment, late lactation cows offered a diet containing corn grain had a greater total DMI and ECM than those offered a diet containing wheat grain. The cows offered the CRN diet had a lower respiration rate, despite greater dry matter intake than cows offered the WHT diet, but there was no difference in the blood biochemical profile. Thus, offering late-lactation dairy cows a diet containing corn in place of wheat may be a useful management tool to aid in the maintenance of dry matter intake and milk production during periods of hot weather because of less heat produced during fermentation and digestion. The economic impact of feeding corn over wheat grain will need to be assessed before comparative values can be determined.

## Figures and Tables

**Table 1:** Composition of main dietary ingredients (g/kg DM unless otherwise stated).

Parameter	Corn	Wheat	Alfalfa
Crude protein	90	136	186
Neutral detergent fiber	63	88	447
Starch	741	616	9
Ash	11.3	22.7	92.8
Crude fat	37	18	16
Gross energy (MJ/kg DM)	18.8	18.5	17.5
Metabolizable Energy (MJ/kg DM)	14.6	13.2	9.0

**Table 2:** Mean intake of total dry matter (kg/day), grain (kg/day), forage (kg/day), metabolizable energy (MJ/day), and starch (kg DMI/day) during each period of the experiment, and on day 7 of the recovery period.

Parameter	CRN ^1^	WHT ^1^	SED ^2^	*p*-Value
n	7	7		
Pre-challenge				
Grain DMI ^3^	6.2	6.2	0.02	0.190
Forage DMI	12.8	12.8	0.04	0.519
Total DMI	19.0	19.0	0.04	0.227
MEI ^4^	206	197	0.4	0.001
Starch	4.7	3.9	0.01	0.001
Heat challenge				
Grain DMI	5.9	5.9	0.07	0.441
Forage DMI	11.5	9.9	0.56	0.016
Total DMI	17.4	15.8	0.57	0.021
MEI	189	167	5.3	0.002
Starch	4.5	3.7	0.05	0.001
Recovery (mean)				
Grain DMI	6.2	6.2	0.02	0.293
Forage DMI	11.7	10.8	0.41	0.042
Total DMI	17.9	17.0	0.44	0.061
MEI	196	179	4.1	0.002
Starch	4.7	3.9	0.02	0.001
Recovery (day 7)				
Grain DMI	6.0	6.0	0.01	0.282
Forage DMI	12.0	12.2	0.31	0.398
Total DMI	18.0	18.2	0.31	0.387
MEI	193.9	197.6	2.76	0.206
Starch	4.6	3.8	0.01	0.000

^1^ CRN = corn-grain based diet, WHT = wheat-grain based diet. ^2^ SED = standard error of the difference. ^3^ DMI = dry matter intake (kg/day) ^4^ MEI = metabolizable energy intake.

**Table 3:** Mean milk yield (kg/day) and composition (g/kg) during each period of the experiment, and on day 7 of the recovery period.

Parameter	CRN ^1^	WHT ^1^	SED ^2^	*p*-Value
n	7	7		
Pre-challenge				
Milk production				
Milk yield	18.2	18.2	0.56	0.997
Energy corrected milk	19.4	18.5	0.71	0.246
Fat	0.82	0.76	0.038	0.173
Protein	0.61	0.59	0.026	0.384
Lactose	0.92	0.89	0.045	0.468
Milk composition				
Fat	44.5	42.7	1.98	0.380
Protein	33.8	32.7	1.19	0.406
Lactose	49.6	49.4	1.17	0.842
Heat challenge				
Milk production				
Milk yield	17.8	16.4	0.82	0.125
Energy corrected milk	19.2	17.6	0.92	0.097
Fat	0.83	0.76	0.048	0.154
Protein	0.59	0.53	0.025	0.040
Lactose	0.92	0.84	0.044	0.098
Milk composition				
Fat	45.8	47.3	2.04	0.474
Protein	33.1	33.0	1.08	0.952
Lactose	51.0	51.6	0.56	0.289
Recovery (mean)				
Milk production				
Milk yield	17.1	15.8	0.79	0.126
Energy corrected milk	18.2	16.3	0.53	0.005
Fat	0.77	0.65	0.030	0.004
Protein	0.58	0.53	0.013	0.005
Lactose	0.86	0.80	0.032	0.062
Milk composition				
Fat	44.9	41.6	2.39	0.191
Protein	34.4	33.2	0.10	0.223
Lactose	50.7	49.3	0.47	0.019
Recovery (day 7)				
Milk production				
Milk yield	18.3	17.1	0.98	0.281
Energy corrected milk	19.0	17.6	1.18	0.248
Fat	0.77	0.72	0.074	0.494
Protein	0.62	0.58	0.031	0.311
Lactose	0.93	0.84	0.048	0.113
Milk composition				
Fat	42.0	43.9	3.3	0.594
Protein	34.8	34.1	1.3	0.585
Lactose	50.2	50.1	0.59	0.956

^1^ CRN = corn-grain based diet, WHT = wheat-grain based diet. ^2^ SED = standard error of the difference.

**Table 4:** Vaginal temperature (VT, °C), and respiration rate (breaths per minute) during each period of the experiment, and on day 6 of the recovery period.

Parameter	CRN ^1^	WHT ^1^	SED ^2^	*p*-Value
n	7	7		
Pre-challenge				
Mean VT	38.4	38.4	0.07	0.806
Minimum VT	37.7	37.8	0.07	0.578
Maximum VT	39.5	39.4	0.09	0.699
Duration VT > 38.8 °C (min)	192	219	43.3	0.557
Respiration rate	34	38	3.4	0.226
Heat challenge				
Mean VT	39.3	39.5	0.13	0.179
Minimum VT	38.4	38.5	0.14	0.875
Maximum VT	40.2	40.3	0.09	0.508
Duration VT > 38.8 °C (min)	1016	1135	110	0.300
Respiration rate	79	93	5.0	0.017
Recovery (mean)				
Mean VT	38.3	38.3	0.05	0.309
Minimum VT	37.7	37.6	0.05	0.287
Maximum VT	39.3	39.1	0.09	0.059
Duration VT > 38.8 °C (min)	142	148	35.6	0.862
Respiration rate	46	55	4.1	0.039
Recovery (day 6)				
Mean VT	38.4	38.3	0.07	0.442
Minimum VT	37.7	37.7	0.08	0.731
Maximum VT	39.1	39.2	0.12	0.570
Duration VT > 38.8 °C (min)	87	156	46.9	0.177
Respiration rate	77	66	7.3	0.146

^1^ CRN = corn-grain based diet, WHT = wheat-grain based diet. ^2^ SED = standard error of the difference.

**Table 5:** Mean blood-gas partial pressures (mmHg) of carbon dioxide (pCO_2_) and oxygen (pO_2_) and blood concentrations (mmol/L) of total carbon dioxide (TCO_2_) and selected analytes.

Parameter	CRN ^1^	WHT ^1^	SED ^2^	*p*-Value
n	7	7		
Pre-challenge				
pCO_2_	77	80	5.3	0.575
pO_2_	82	80	26.2	0.925
TCO_2_	2	30	1.1	0.376
Bicarbonate ^3^	27	28	1.0	0.372
Sodium ^4^	132.5	132.4	0.79	0.877
Potassium ^5^	12.0	12.1	0.1	0.073
Chloride ^6^	106	106	1.6	0.816
Glucose	3.3	3.5	0.10	0.204
Lactate	0.78	1.18	0.159	0.039
Heat challenge				
pCO_2_	69	62	3.9	0.145
pO_2_	108	107	22.9	0.952
TCO_2_	25	24	0.8	0.835
Bicarbonate	23	23	0.7	0.993
Sodium	134.5	135.7	0.62	0.081
Potassium	11.5	11.4	0.48	0.753
Chloride	111	110	1.0	0.194
Glucose	3.6	3.7	0.11	0.286
Lactate	1.44	1.43	0.254	0.975
Recovery (mean)				
pCO_2_	77	77	7.2	0.956
pO_2_	144	106	33.0	0.267
TCO_2_	27	28	1.0	0.561
Bicarbonate	25	26	0.9	0.509
Sodium	132.3	132.4	1.05	0.940
Potassium	11.8	12.1	0.17	0.093
Chloride	108	107	1.5	0.336
Glucose	3.4	3.5	0.16	0.159
Lactate	0.47	0.76	0.194	0.168

^1^ CRN = corn-grain based diet, WHT = wheat-grain based diet. ^2^ SED = standard error of the difference. ^3^ Bicarbonate = HCO_3_^−^. ^4^ Sodium = Na^+^. ^5^ Potassium = K^+^. ^6^ Chloride = Cl^−^.

**Table 6:** Mean change per day (mean regression slope) within the heat challenge and recovery periods for feed intake, milk, and vaginal temperature parameters.

Parameter	CRN ^1^	WHT ^1^	SED ^2^	*p*-Value
Heat challenge				
Feed intake (kg/day)				
Grain DMI ^3^	−0.08	−0.12	0.035	0.323
Forage DMI	−0.51	−1.31	0.334	0.034
Total DMI	−0.60	−1.43	0.341	0.031
Milk production (kg/day)				
Milk yield	−0.53	−0.84	0.337	0.365
ECM ^4^	−0.43	−0.64	0.345	0.559
Fat	−0.01	−0.01	0.014	0.974
Protein	−0.02	−0.03	0.011	0.508
Lactose	−0.03	−0.05	0.018	0.348
Milk composition (g/kg)				
Fat	0.02	0.14	0.047	0.025
Protein	−0.05	−0.05	0.027	0.157
Lactose	0.04	0.01	0.022	0.157
Vaginal temperature (°C)				
Mean	0.34	0.36	0.063	0.822
Minimum	0.27	0.23	0.054	0.465
Maximum	0.31	0.24	0.070	0.343
Duration > 38.8 °C (min)	272	230	50.3	0.420
Recovery				
Feed intake (kg/day)				
Grain DMI	0.00	0.00	0.005	0.830
Forage DMI	0.15	0.42	0.110	0.029
Total DMI	0.17	0.42	0.119	0.056
Milk production (kg/day)				
Milk yield	0.44	0.81	0.186	0.074
ECM	0.53	0.69	0.121	0.225
Fat	0.02	0.02	0.007	0.892
Protein	0.02	0.03	0.005	0.025
Lactose	0.02	0.04	0.004	0.003
Milk composition (g/kg)				
Fat	0.02	−0.02	0.035	0.312
Protein	0.02	0.03	0.013	0.614
Lactose	−0.01	0.00	0.010	0.345
Vaginal temperature (°C)				
Mean	0.05	0.06	0.021	0.756
Minimum	0.05	0.10	0.038	0.214
Maximum	0.04	−0.01	0.028	0.116
Duration >38.8 °C (min)	2.2	0.6	15.1	0.920

^1^ CRN = corn-grain based diet, WHT = wheat-grain based diet. ^2^ SED = standard error of the difference. ^3^ DMI = dry matter intake ^4^ ECM = energy corrected milk.

## Data Availability

Data are available upon reasonable request to the corresponding author.
